# Exploring the Link Between Tinnitus and Loudness Intolerance *

**DOI:** 10.1055/s-0046-1819562

**Published:** 2026-05-20

**Authors:** Agnes Yohannan, Archana Gundmi

**Affiliations:** 1 Department of Speech and Hearing, Manipal College of Health Professions, Manipal Academy of Higher Education, Manipal, India

**Keywords:** tinnitus, hyperacusis, loudness intolerance, loudness discomfort

## Abstract

**Introduction:**

A ringing sensation in the ear is referred to as tinnitus. It is one of the common symptoms associated with hearing loss. It can be associated even with loudness-related conditions, such as hyperacusis.

**Objective:**

To investigate the relationship between tinnitus and hyperacusis in individuals with normal hearing and up-to-mild hearing loss, focusing on the manifestation of loudness intolerance.

**Methods:**

The Tinnitus Handicap Inventory (THI) was administered among 35 adults having primary complaints of tinnitus with normal hearing and up-to-mild hearing loss. Further, the Modified Khalfa Hyperacusis Questionnaire (M-HQ) was used to evaluate the hyperacusis severity among the same individuals. Spearman's correlation coefficient was used to check the correlation between tinnitus and hyperacusis severity.

**Results:**

Of the 35 adults who reported tinnitus, 18 (51.4%) of them reported having hyperacusis. The outcome of Spearman's correlation indicated a moderate positive correlation between the THI and M-HQ scores r _s_ = 0.401( *p* = 0.017).

**Conclusion:**

The results obtained suggest that tinnitus is associated with hyperacusis. With the subjective evaluation, it is clear that the change in the severity of tinnitus perception is interconnected with the perception of hyperacusis severity. But this relationship is not a completely overlapping one. Future research on the comorbid interplay of tinnitus severity and hyperacusis severity may shed further light on this link.

## Introduction

Tinnitus is the sensation of ringing, humming, or whistling sounds in the absence of external sensory stimuli. 1 According to the World Health Organization, tinnitus affects 278 million people worldwide, or about 15% of the global population, with a prevalence rate of about 10% in adults. 2 Tinnitus can create a significant impact on a person's social, emotional, and other aspects of daily living. Adrian and El Refaie 1 demonstrated that 2.8% of people reported tinnitus to be moderately annoying, while 1.6% reported it to be severely annoying. Tinnitus was said to have a severe impact on the desire to live a regular life in 0.5% of people. Tinnitus can be associated with many other pathologies like hearing loss and hyperacusis, which can further intensify the negative impact in an individual's life. 3 The estimated prevalence of hyperacusis in individuals with tinnitus is 55 to 86%. 4 5 6 7 Hyperacusis is characterized by a reduced tolerance to everyday sounds that are uncomfortably painful or loud, limiting social, occupational, and recreational activities. 8

Hébert et al. 9 postulated that tinnitus and hyperacusis share associated pathophysiology. Accordingly, tinnitus is due to elevated spontaneous activity along the auditory pathway, whereas hyperacusis occurs due to elevated stimulus-related activity. This common pathophysiology was supported by the neural gain model. 10 11 According to this model, tinnitus could emerge in an individual with hearing loss as a failure to adapt the missing sensory information, which results in an auditory enhancement. 12 13 14

Literature has shown evidence for this association clinically, through various objective as well as subjective measures of tinnitus and hyperacusis. 6 15 16 17 18 Gu et al. 13 used functional magnetic resonance imaging (MRI) to measure sound-evoked activation of central auditory centers. This study showed an elevated activation in the primary auditory cortex for those with tinnitus as well as hyperacusis. The findings demonstrate that hyperacusis and tinnitus are linked to hyperactivity in the central auditory system, showing their common pathophysiological relationship. A study by Raj-Koziak et al. 17 shows hyperacusis scores were positively correlated with Tinnitus Handicap Inventory (THI) scores, indicating that the more severe the hyperacusis, the more severe the tinnitus was.

Tinnitus and hyperacusis are purely subjective phenomena. 20 Hence, it is appropriate to use subjective measures, to quantify individualized tinnitus and hyperacusis symptoms and their severity. Since both conditions share similar pathophysiology and clinical symptoms, for a thorough understanding of each disorder's impact, it is appropriate to include hyperacusis evaluation in individuals with tinnitus. 15 Also, in the assessment of hyperacusis, it is suitable to have the tinnitus evaluation as well. There is a dearth of literature that encompasses this complete profile of evaluation among these disorders. The current study included the subjective questionnaires THI, to determine the perceived tinnitus, and Modified Khalfa Hyperacusis Questionnaire (M-HQ) for understanding the subjective perception of uncomfortable loudness. This kind of evaluation helps to create appropriate assessment protocol as well as rehabilitation plans for these disorders. Therefore, the current study used self-reported questionnaires to assess hyperacusis in people with various degrees of tinnitus, who had tinnitus as their primary complaint.

## Methods

### Approval

Approval for the current study was obtained from the Institutional Research Committee (IRC), Institutional Ethics Committee (IEC) (IEC No: IEC228/2020), and Clinical Trial Registry of India (CTRI) (CTRI/2020/07/026309) before the recruitment of participants for the study. Permission from the respective authors of the THI and M-HQ has also been obtained to be used in the current study.

### Participants

The present study included 35 adults (21 men and 14 women) aged 21 to 65 years (M = 42.89 ± 14.67) who presented to the Department of Speech and Hearing for audiological evaluation. To be eligible for participation, individuals should have normal hearing or hearing loss or only an up-to-mild degree of hearing loss (up to 40 dBHL) in both ears. Participants should also have tinnitus as the primary complaint and must have a THI score >18. Written consents or online mode of consent was obtained from all the research participants before the questionnaire administration. Participants did not receive financial compensation for their participation.

### Measures

The following instruments were used for the study:

### Tinnitus Handicap Inventory (THI)

The THI is a self-reported tool for calculating the effect of tinnitus on everyday life. 20 The THI consists of 25 items divided into 3 subscales: emotional, functional, and catastrophic, with answer options such as *no* (0 points), *sometimes* (2 points), and *yes* (4 points). Tinnitus is classified as no handicap, mild, moderate, severe, or catastrophic handicap based on the scoring, ranging from 0 to 100. The THI score categories are defined as follows: a score of 0 to 16 corresponds to “no or slight handicap”, 18 to 36 indicates “mild”, 38 to 56 indicates “moderate”, 58 to 76 indicates “severe”, and a score falling within the range of 78 to 100 is classified as “catastrophic handicap”.

### Modified Khalfa et al.'s 21 Hyperacusis Questionnaire (M-HQ)

The M-HQ assesses the subjective distress related to hypersensitivity to sound based on 20 items. 21 It has 3 domains: functional, social, and emotional, with answer options of *no* (0 points), *sometimes* (3 points), and *yes* (5 points). The overall scores vary from 0 to 100, which classifies hyperacusis into normal, mild, moderate, or severe hyperacusis.

### Procedure

#### Administration of Questionnaires

At first, participants were first asked to fill the THI. 20 In the offline mode of administration, the questionnaire was directly handed out by instructing the participant about the choice of response. Due to the coronavirus disease (COVID-19) pandemic happening at the time, some of the participants completed their response online (social media, emails).

After the THI administration, the total score of the THI was calculated by the clinician. Further, individuals having THI scores greater than 18 (mild degree) were only considered for administration of the M-HQ. 21 This questionnaire was administered in a similar way as the previous questionnaire, either presential or online.

### Statistical Analysis

For quantitative data, descriptive statistics were calculated, and for qualitative data, percentages were calculated. The histogram was used to determine the distribution of total THI and M-HQ ratings. The data were checked for normality using the Shapiro-Wilk test, which indicated non-normal distribution. Hence, the relationship between tinnitus and hyperacusis was evaluated with the Spearman correlation coefficient. The analysis was performed using IBM SPSS Statistics for Windows (IBM Corp.) software, version 27.0.

## Results

The mean age of the participants was of 42.89 ± 14.67. Table 1 displays the frequency distribution of participants based on gender.

**Table 1:** Gender distribution of patients who reported tinnitus as their primary complaint

	Frequency	Percentage	Valid percentage	Cumulative percentage
Female	14	40.0	40.0	40.0
Male	21	60.0	60.0	100
Total	35	100.0	100.0	

### Tinnitus Assessment

The present study used THI for the evaluation of tinnitus. Table 2 represents the descriptive statistics of THI. In the current study, upon THI analysis of the participants, it was found that 19 individuals (54.3%) experienced mild tinnitus, 11 (31.4%) had moderate tinnitus, 3 (8.6%) reported severe tinnitus, and 2 (5.7%) suffered from catastrophic tinnitus. The frequency distribution of severity is provided in Table 3 .

**Table 2:** Descriptive statistics for Tinnitus Handicap Inventory scores

Mean	Median	Standard deviation	Interquartile range	
37.20	32.00	18.681	18–82	

**Table 3:** Frequency distribution of Tinnitus Handicap Inventory severity

Tinnitus Handicap Inventory impression	Frequency	Percent	Cumulative percentage
Mild	19	54.3	54.3
Moderate	11	31.4	85.7
Severe	3	8.6	94.3
Catastrophic	2	5.7	100.0
Total	35	100.0	

### Hyperacusis Assessment

In the present study, the M-HQ was utilized for hyperacusis evaluation. Among the study participants, 18 individuals (51.4%) reported experiencing hyperacusis, with 11 having mild hyperacusis (31.4%), 4 exhibiting moderate hyperacusis (11.4%), and 3 indicating severe hyperacusis (8.6%). Descriptive statistics for the M-HQ scores are shown in Table 4 . The frequency distribution of severity is given in Table 5 .

**Table 4:** Descriptive statistics for Modified Khalfa Hyperacusis Questionnaire scores

Mean	Median	Standard deviation	Interquartile range	
20.09	12.00	23.513	0–86	

**Table 5:** Frequency distribution of Modified Khalfa et al.'s 21 Hyperacusis Questionnaire severity

M-HQ Impression	Frequency	Percentage	Cumulative percentage
Normal	17	48.6	48.6
Mild	11	31.4	80.0
Moderate	4	11.4	91.4
Severe	3	8.6	100.0
Total	35	100.0	

### Relationship between Tinnitus and Hyperacusis

Among the current study participants, 18 (51.42%) had hyperacusis as an associated symptom. The frequency distribution of hyperacusis occurrence in patients with varying degrees of tinnitus is shown in Table 6 .

**Table 6:** Frequency distribution of hyperacusis severity in individuals with tinnitus

Tinnitus	Hyperacusis			
	Normal	Mild	Moderate	Severe
Mild	11 (57.9%)	7 (36.8%)	–	1 (5.3%)
Moderate	5 (45.5%)	3 (27.3%)	3 (27.3%)	–
Severe	–	1 (33.3%)	1 (33.3%)	1 (33.3%)
Catastrophic	1 (50%)	–	–	1 (50%)

Table 7 reveals Spearman's correlation coefficient to demonstrate the relationship between the severity of tinnitus and hyperacusis.

**Table 7:** Relationship between tinnitus and hyperacusis severity using the Spearman's correlation coefficient

			THI	M-HQ
Spearman's rho	THI	Correlation coefficient	1.000	0.401*
		Significance (2-tailed)	–	0.017
		N	35	35
	M-HQ	Correlation coefficient	0.401*	1.000
		Significance (2-tailed)	0.017	–
		N	35	35

**Abbreviations** : M-HQ, Modified Khalfa et al.'s 21 Hyperacusis Questionnaire; THI, Tinnitus Handicap Inventory.

**Note** : *Correlation is significant at the 0.05 level (2-tailed).

The outcome of Spearman's correlation indicated a moderate positive correlation between the THI and M-HQ scores r _s_ = 0.401 ( *p* = 0.017). The guidelines provided by Akoglu 22 were used to interpret the correlation findings (the interpretation of the Pearson's and Spearman's correlation coefficients r _s_ score +0.4 −0.4 indicates moderate correlation).

## Discussion

### Tinnitus Assessment

A total of 35 participants having primary complaints of tinnitus have been included in the study. The mean score obtained in the THI was 37.20. This indicates that, most of the participants presented a mild degree of tinnitus on the THI severity scale. There is a similar study that reported a mean score of 35. 24 Raj-Koziak et al. 17 17 19 showed a mean score of 58.9, Aazh et al. 19 26 showed a mean score of 44.7, and Degeest et al. 19 reported a mean score of 44.2. This kind of discrepancy in mean scores can be explained by the variations in number of participants and the reduced representation of participants in the higher severity of the disorder.

### Hyperacusis Assessment

The current study used the M-HQ for the measurement of hyperacusis. Among the 35 people in this study who reported tinnitus as their primary complaint, 17 (48.6) of them reported normal scores, and 18 (51.4%) reported having hyperacusis. This indicates that the majority of the current study participants had hyperacusis. The majority of the earlier studies used the Hyperacusis Questionnaire (HQ) for the evaluation, and they also reported that most of the participants had hyperacusis. 25

### Correlation: Tinnitus Severity and Hyperacusis

The prevalence of hyperacusis in individuals with tinnitus was reported to be almost 55 to 86%. 4 5 6 7 This highlights the comorbid relationship between these disorders. Hence, it is crucial to understand the direction of this relationship. The Spearman's correlation coefficient between tinnitus and hyperacusis severity from this study was r = 0.401. This indicates a moderate correlation between the severity of disorders. This demonstrates that the perception of hyperacusis severity changes as the tinnitus severity changes. Similar findings were observed in the previous literature. Raj-Koziak et al. 17 found an association of r = 0.44 between hyperacusis and tinnitus severity for HQ and THI ratings. Fackrell et al. 23 got a similar result, r = 0.49. A study by Raj-Koziak et al. 17 also showed a correlation of r = 0.5, with THI and HQ. They arrived at the conclusion that hyperacusis could exacerbate the severity of perceived tinnitus. Few studies also affirm a stronger correlation between these disorders. Cederroth et al. 24 demonstrated that hyperacusis is strongly associated with tinnitus, and the relationship increased with higher tinnitus severity.

The variation in the relationship can be accounted for by the disproportionate representation of various severities of the disorder.

Thus, it can be stated that there exists a large overlap in prevalence between tinnitus and hyperacusis. Since the pathophysiological mechanisms also show a shared mechanism, this kind of overlap is observable between the disorders. Clinically, this interconnection is evident with various objective and subjective measures. It is understood that, according to the changes in the category of tinnitus, there is a significant change in the perception of loud sounds. These results can be directly linked with the previous functional MRI studies that showed hyperactivity in the central auditory system for tinnitus and hyperacusis, indicating their common pathophysiological relationship and their common functional mechanism.

There also exist different thoughts and evidence related to tinnitus and hyperacusis that have shown no correlation between tinnitus and hyperacusis severity. According to Magalhães et al., 25 there was no direct relationship between hyperacusis and tinnitus severity. Dauman and Bouscau-Faure 5 used a scale of 0 to 10 to measure the discomfort of tinnitus and the total annoyance of hyperacusis and found that the association between tinnitus and hyperacusis annoyance was weak (r = 0.35). There are even studies that show the different neural activity for these disorders in the auditory pathway, specifically in the cingulate and orbitofrontal cortex. 13 Thus, it can be extrapolated that, despite their similarities, they are not arising from the same mechanism.

With the current study findings of moderate correlation, it cannot be substantially stated the relationship between tinnitus and hyperacusis is entirely a linear one. Hence it can be assumed that, even though the pathophysiology and symptoms are similar, which shows shared underlying mechanisms, there is no complete overlap between these two disorders.

### Implications for Diagnosis and Treatment

The co-occurrence of tinnitus and hyperacusis has significant diagnostic and treatment implications. The current study shows the importance to evaluate both tinnitus and hyperacusis in case of its comorbid presence. As both coexist, this may intensify the negative impact on quality of life. The determination of which symptom appeared first, and which is their main complaint should be used in the formulation of treatment plan.

### Future Implication

The present study sheds light on the extent of the relationship between tinnitus and hyperacusis. However, it is important to note that more research is needed to understand the fundamental causes that lead to these conditions. While preclinical hearing loss may be a potential risk factor, the psychological aspects of tinnitus and hyperacusis also warrant thorough investigation.

Furthermore, longitudinal studies with a large sample size can help determine the direction of the association between these disorders more effectively. Such studies not only provide insight into the treatment progress in individuals with concurrent tinnitus and hyperacusis but also offer valuable data for understanding the underlying mechanisms. Its findings may be limited by factors such as sample size, study design, and potential confounding variables. Therefore, future research should aim to address these limitations for a more comprehensive understanding of these conditions.

## Conclusion

From the current study, it can be concluded that tinnitus can be associated with hyperacusis but not completely overlapping. According to the change in the category of tinnitus, there is a significant shift in the perception of loud sounds. Future studies investigating this comorbid interaction of tinnitus and hyperacusis severity may provide additional insights into this relationship.
